# Preclinical Pharmacological Actions of Alpinetin and Pinocembrin—A Comparative Review

**DOI:** 10.3390/ph19050734

**Published:** 2026-05-07

**Authors:** Xinxiang Chen, Siu Kan Law, Huajian Li, Mei Zhang, Wenying Yu, Yixiao Li, Ying Zhou, Albert Wing Nang Leung, Bo Wu, Chuanshan Xu, Mei Feng

**Affiliations:** 1 Guangzhou Municipal and Guangdong Provincial Key Laboratory of Molecular Target & Clinical Pharmacology, The NMPA and State Key Laboratory of Respiratory Disease, School of Pharmaceutical Sciences, Guangzhou Medical University, Guangzhou 511436, China; 2023211626@stu.gzhmu.edu.cn (X.C.); zhmeic@gzhmu.edu.cn (M.Z.); 2021123056@stu.gzhmu.edu.cn (W.Y.); 2021123055@stu.gzhmu.edu.cn (Y.L.); 2021123029@stu.gzhmu.edu.cn (Y.Z.); wubo@gzhmu.edu.cn (B.W.); 2 Independent Researcher, Hong Kong SAR, China; siukanlaw@hotmail.com; 3 The Affiliated Panyu Central Hospital of Guangzhou Medical University, Guangzhou 511400, China; 2022211570@stu.gzhmu.edu.cn; 4 School of Graduate Studies, Lingnan University, Tuen Mun, Hong Kong SAR, China; albertleung@ln.edu.hk

**Keywords:** traditional Chinese medicine, flavonoids, alpinetin, pinocembrin, anti-inflammatory, anti-tumor, antibacterial

## Abstract

**Background**: Human diseases remain a major global health challenge, requiring effective therapeutic strategies. Traditional Chinese medicine (TCM) has been widely used in clinical settings. Many natural compounds, such as flavonoids from TCM, exhibit diverse pharmacological activities. Alpinetin and pinocembrin are structurally related flavonoids. Alpinetin is derived from *Zingiberaceae* plants, and pinocembrin is extracted from wild marjoram (origanum vulgare) or other natural sources. They possess a wide range of pharmacological activities or biological effects, including anti-inflammatory, anti-tumor, liver and kidney protection, cardiovascular protection, and antibacterial activities. **Methods**: The present comparative review was conducted in accordance with the Preferred Reporting Items for Systematic Reviews and Meta-Analyses (PRISMA) guidelines, using four major databases (PubMed, EMBASE, Web of Science, and Cochrane Library), as well as CNKI without language restrictions. **Results**: Pharmacokinetic studies reveal distinct absorption, metabolism, and excretion profiles. Alpinetin and pinocembrin undergo glucuronidation and interact with cytochrome P450 enzymes and transporters. However, alpinetin has demonstrated approximately 1.5-fold higher plasma exposure and slower clearance compared to pinocembrin. Mechanistically, alpinetin exerted therapeutic effects through modulation of the NF-κB/MAPK, PI3K/Akt, and PPAR-γ signaling pathways, resulting in a 2- to 3-fold reduction in pro-inflammatory cytokines. In contrast, pinocembrin exerted protective activity through the inhibition of HMGB1/TLR4 signaling, regulation of endoplasmic reticulum stress, and activation of Nrf2/HO-1, leading to a 1.8-fold increase in antioxidant enzyme activity. The minimum inhibitory concentrations were reduced by 2- to 4-fold against Gram-positive bacteria compared to alpinetin. **Conclusions**: These findings highlight the pharmacological potential of alpinetin and pinocembrin as promising candidates for the development of novel anti-tumor, anti-inflammatory, liver and kidney protection, cardiovascular protection, and antibacterial agents. However, research on the pharmacological actions of alpinetin and pinocembrin is still in the preclinical stage. Further research is required to validate their efficacy in clinical settings, especially for translation to clinical studies. This is critical to translating these natural flavonoids into effective therapeutic agents while addressing the regulatory challenges and pathways associated with botanical drugs in human diseases.

## 1. Introduction

Traditional Chinese medicine (TCM) plays a significant role in disease treatment and management, providing a holistic framework including preventive measures, personalized treatment, and care [1]. Health maintenance in TCM relies on the harmony of “yin” and “yang”, “qi” (vital energy), and the five elements [2]. TCM integrates complementary herbal remedies to address the root cause of illness [3], which emphasizes holistic regulation through natural compounds and multi-herbal formulations [4]. In these herbal or multi-component formulations, flavonoids are often considered the major bioactive compounds contributing to therapeutic effects [5].

**Figure 1:**
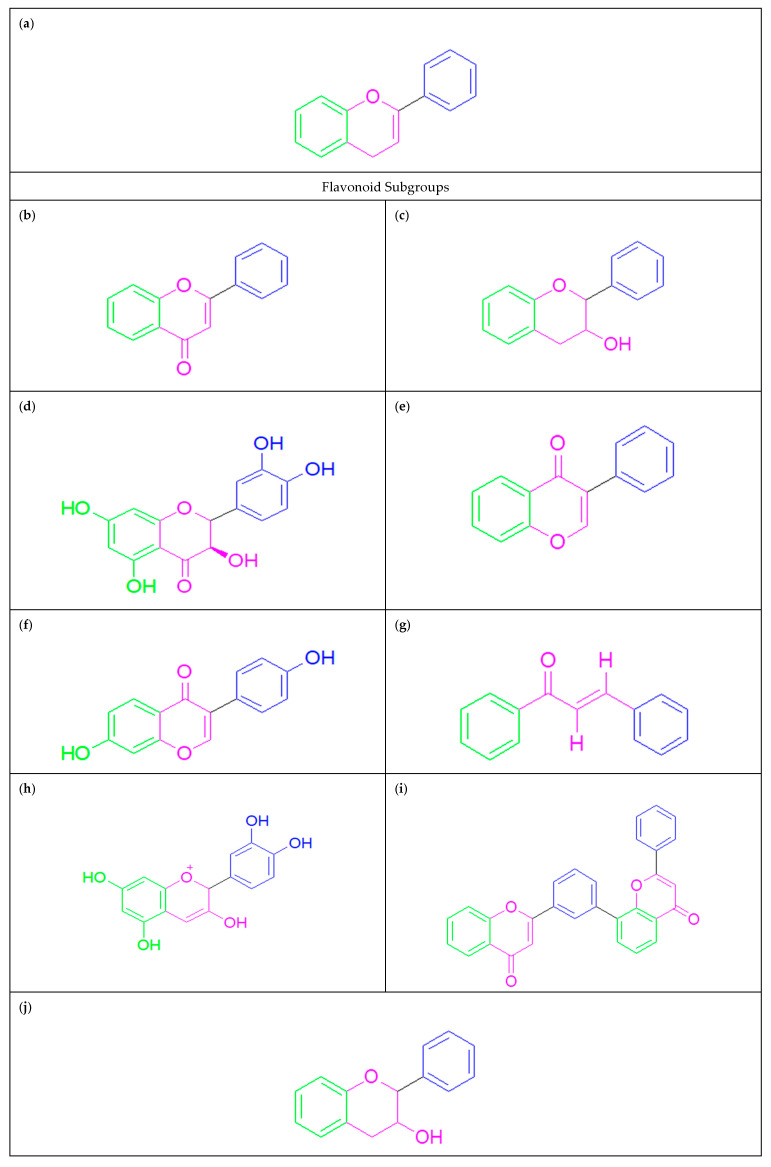
Chemical structures of (**a**) Flavonoids (C6–C3–C6 skeleton), (**b**) Flavones (Double bond at C2–C3 and carbonyl group at C4), (**c**) Flavonols (Flavone and hydroxyl group at C3), (**d**) Dihydroflavonols (Saturated C2–C3 bond and hydroxyl groups at C3), (**e**) Isoflavones (Benzene ring attached at C3 instead of C2), (**f**) Dihydroisoflavones (Isoflavone with saturated C2–C3 bond), (**g**) Chalcones (Open-chain α,β-unsaturated carbonyl system), (**h**) Anthocyanins (Flavylium cation core), (**i**) Biflavones (Two flavone units linked together), and (**j**) Flavanols (Saturated C2–C3 bond, hydroxyl group at C3, no carbonyl group at C4).

“Flavonoids” are a broad class of naturally occurring compounds derived from 2-phenylchroman-4-one. They generally consist of two benzene rings linked through a three-carbon bridge forming a C6–C3–C6 skeleton [6]. These compounds are widely distributed in the plant kingdom and exhibit considerable structural diversity [7]. Based on their specific chemical frameworks, flavonoids are classified into several subgroups, including flavones [8], flavonols [9], dihydroflavonols [10], isoflavones [11], dihydroisoflavones [12], chalcones [13], anthocyanins [14], biflavones [15], flavanols [16], and so on (Figure 1). Previous studies have demonstrated that flavonoids possess a wide range of pharmacological activities, such as anti-inflammatory effects [17], anti-tumor activity [18,19], cardiovascular protection [20], and prevention and treatment of peptic ulcers [21], as well as weight management [22]. A recent study revealed the potential for combating COVID-19 [23]. Among the thousands of flavonoids, alpinetin and pinocembrin have a unique structural simplicity–activity relationship and share a structurally simple backbone, which has demonstrated favorable safety and tolerability in preclinical models. They have consistently remained a major focus in drug discovery and development against human diseases [24].

**Figure 2:**
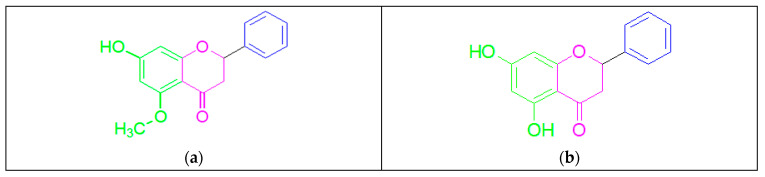
Chemical structures of (**a**) alpinetin and (**b**) pinocembrin.

“Alpinetin” and “pinocembrin” are naturally occurring flavonoids. They exhibit a close biosynthetic relationship, arising from common precursors and enzymatic pathways, a connection that reflects their chemical structural similarity [25]. Alpinetin is isolated from the seeds of *Zingiberaceae* plants, and its chemical structure has been identified as 7-hydroxy-5-methoxydihydroflavone. Its distribution extends beyond *Zingiberaceae* species to encompass a wide range of dicotyledons [26]. Alpinetin is a major constituent of cardamom seeds, although different *Zingiberaceae* plants contain diverse chemical compositions [27]. Pinocembrin is a precursor of alpinetin, extracted from *Wild marjoram* (*Origanum vulgare*), *honey*, *propolis*, and *ginger roots*, with the chemical structure 5,7-dihydroxyflavanone (Figure 2) [28].

Alpinetin and pinocembrin are included in the Chinese Materia Medica and are derived from members of the *Zingiberaceae* (ginger) family, which has a long history of medicinal use, such as *Alpinia japonica* (Thunb.) Miq., *Elettaria cardamomum* (L.) Maton, *Alpiniae katsumadai* Hayata, and Galangae fructus. The latter two also exist in the Chinese pharmacopoeia. More recently, alpinetin and pinocembrin, possessing the characteristic flavonoid backbone with a wide range of pharmacological activities, have attracted considerable scientific attention. Notably, these compounds are naturally derived from plants, exhibit low toxicity, and have long been utilized in traditional Chinese medicine for therapeutic purposes.

This review contains a comparative and integrative analysis of alpinetin and pinocembrin. It is different from previous reviews, which separately summarized the pharmacology and pharmacokinetics of these flavonoids, systematically analyzing their absorption, distribution, metabolism, and excretion. NFκB/MAPK, PI3K/Akt, and PPARγ are the major signaling pathways of alpinetin, while pinocembrin has the HMGB1/TLR4, ER stress, and Nrf2/HO1 signaling pathways. Alpinetin shows a 2- to 3-fold reduction in cytokines compared to pinocembrin’s 1.8-fold increase in antioxidant enzyme activity. This emphasizes differential drug–drug interaction risks through CYP450 and transporter inhibition. The translational insights for clinical application, across multiple models, such as respiratory, digestive, cardiovascular, hepatic, and renal diseases, are discussed. These integrate pharmacokinetics, pharmacodynamics, and mechanistic differences and contribute to the broader framework of botanical drug development.

## 2. Search Strategy

**Figure 3:**
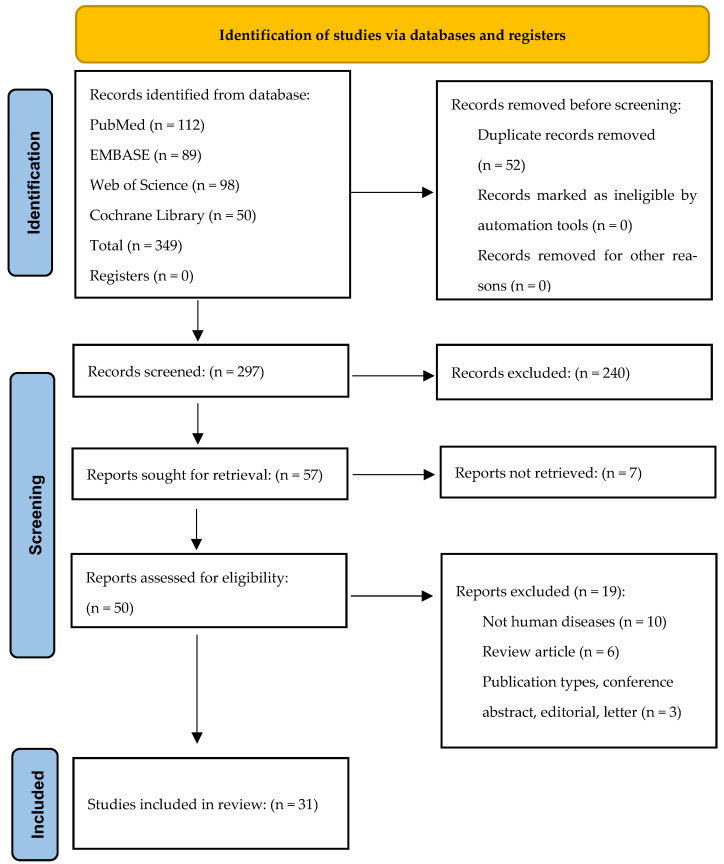
Systematic Reviews and Meta-Analyses (PRISMA) guidelines.

A comparative review was conducted in accordance with the Preferred Reporting Items for Systematic Reviews and Meta-Analyses (PRISMA) guidelines (Figure 3). This consisted of a search strategy across four major databases, PubMed, EMBASE, Web of Science, and Cochrane Library, focusing on the investigation of pharmacological properties, mechanisms of action, and therapeutic potential of flavonoids, particularly alpinetin and pinocembrin, in the context of human diseases. The search terms “Therapeutic potential”, OR “Alpinetin”, OR “Pinocembrin”, OR “Respiratory Diseases”, OR “Digestive Diseases”, OR “Reproductive Diseases”, OR “Locomotor Diseases”, OR “Cardiovascular Diseases”, OR “Liver Disease”, OR “Kidney Diseases”, etc., were used as keywords to search all databases for publications within the past twenty years. Two independent reviewers screened titles/abstracts and full texts against eligibility criteria. Risk of bias is summarized narratively and in tabular form. Inclusion criteria included predominantly preclinical studies. Exclusion criteria included studies not related to human diseases, review articles, and publication types such as conference abstracts, editorials, and letters. The study findings are presented and summarized without any language restrictions.

## 3. Pharmacokinetics of Alpinetin and Pinocembrin

Variations in herbal medicine-metabolizing enzymes and pharmacological targets may significantly influence herbal efficacy [29]. Consequently, pharmacokinetic research on traditional Chinese medicines has become essential for achieving clinical application. Pharmacokinetic studies encompass absorption, distribution, metabolism, and excretion (ADME), which collectively describe how the body processes administered substances, including flavonoids such as alpinetin (CAS No.: 36052-37-6; IUPAC Name: (2S)-7-Hydroxy 5-methoxy-2-phenyl-2,3-dihydro-4H-chromen-4-one) and pinocembrin (CAS No.: 480-39 7; IUPAC Name: 5,7-Dihydroxy-2-phenyl-2,3-dihydro-4H-chromen-4-one) [30,31].

“Absorption” occurs mainly in the small intestine following oral administration. The extent and site of intestinal absorption are influenced by multiple physicochemical and physiological factors, like solubility, lipophilicity, luminal concentration, pKa value, transporter substrate specificity, transporter expression, luminal fluid pH, gastrointestinal transit time, and intestinal metabolism [32]. After alpinetin or pinocembrin is absorbed from the site of administration, its “distribution” to extracellular fluids depends on blood plasma and tissue binding, as well as the lipophilicity of the flavonoid [33]. The liver is a major site for “metabolism” and contains numerous metabolic enzymes. This glucuronidation reaction is catalyzed by UDP-glucuronosyltransferases [34] in liver microsomes, representing a major pathway for the inactivation and elimination of diverse exogenous or endogenous compounds. The kidney is the main route for the “excretion” of flavonoids, which involves renal excretion, glomerular filtration, passive tubular reabsorption, active tubular secretion, and urinary elimination [35].

## 4. Absorption and Distribution

A UHPLC-ESI-MS/MS method and micellar electrophoresis possessed higher sensitivity than HPLC for determining the content of alpinetin in rat plasma and calculating its pharmacokinetic parameters. Rats were given cardamom extract (containing 5 mg/kg alpinetin) after oral administration. The dosage of alpinetin over time was established, and the pharmacokinetic parameters were calculated by a non-atrioventricular model. The results showed that the Cmax was 385.633 ± 91.192 ng/mL, T1/2 was 1.5784 ± 0.239 h, AUC (o-t) was 911.723 ± 59.208 ng/mL/h, Vz/F was 24.295 ± 6.858 L/kg, and CLz/F was 10.6834 ± 0.684 L/h/kg [36].

A sensitive and accurate UPLC-ESI-MS/MS method was developed and validated to quantitatively detect pinocembrin-7-O-β-D-glucoside (PCBG) in rat blood plasma and was used to study oral and intravenous administration for the pharmacokinetic parameters. Rats were administered 40 mg/kg of PCBG. The results showed that the Cmax was 109.0 ng/mL, T1/2 was 2.5 ± 0.0 h, AUC (o-t) was 137.6 ng/mL/h, Vz/F was 12.3 L/kg, and CLz/F was 3.4 L/h/kg (Table 1) [37].

## 5. Metabolism

The metabolic spectrum of alpinetin in rats was elucidated by UHPLC-TOF-MS. A comprehensive ion chromatography strategy, incorporating multiple prototype and intermediate metabolite templates, was employed to conduct systematic metabolic profiling. A total of 71 canonical metabolic reactions were incorporated to achieve systematic and comprehensive metabolite analysis. Fifteen compounds were identified in the urine, plasma, bile, and feces of rats given alpinetin by oral administration via the metabolite spectrum analysis strategy. Prototypes, glucuronic acid conjugates, and phenolic acid metabolites may be the main forms of alpinetin in rats. This study systematically clarified the possible metabolic pathway of alpinetin in vivo and provided meaningful information for subsequent pharmacological research [38]. Following glucuronidation, flavonoid metabolites may exhibit either enhanced pharmacological activity compared to the parent compound or entirely distinct biological and pharmacological properties.

In the case of alpinetin, glucuronidation primarily yields oxygen-monoglucuronidated derivatives. The UDP-glucuronosyltransferase (UGT) isoforms implicated in this process consist of UGT1A1, UGT1A3, UGT1A9, and UGT2B15 [39]. Genetic polymorphisms in glucuronosyltransferases significantly influence flavonoid metabolism [40]. Patients with different genotypes may experience variable pharmacokinetic profiles when administered alpinetin.

Cytochrome P450 (CYP450) is a kind of liver microsomal enzyme with catalytic functions such as oxidation, epoxidation, hydroxylation, and demethylation. This is an important metabolic pathway for flavonoids [41]. Since CYP450 enzymes are central to flavonoid metabolism, alpinetin appears to undergo limited CYP-mediated biotransformation, likely with inhibitory interactions with specific isoforms [42]. The transcription of CYP3A4 was significantly upregulated by 2.28 and 1.65 times when induced by 10 μmol/L and 20 μmol/L alpinetin through activation of the pregnane X receptor and CYP3A4 mRNA expression, respectively [43]. Subsequently, alpinetin was incubated separately with the substrates of CYP1A2, CYP3A4, CYP2E1, CYP2D6, CYP2A6, CYP2C8, and CYP2C9. It was identified that alpinetin selectively inhibited the activity of CYP1A2 but had no inhibitory effects on CYP3A4, CYP2C8, or CYP2C9 [44]. Alpinetin may influence CYP3A4-mediated metabolism, potentially reducing the blood concentrations of drugs such as simvastatin and atorvastatin. The competitive inhibition of CYP1A2 can theoretically elevate levels of drugs, including theophylline and warfarin. However, these observations are based on in vitro CYP inhibition assays and may not necessarily translate into clinically significant drug–drug interactions in vivo. Pharmacokinetic and clinical studies are required to determine the actual relevance of these interactions.

Compared with alpinetin, the metabolic spectrum of pinocembrin in rats was elucidated by HPLC-DAD. Pinocembrin inhibited the metabolic activity of cytochrome P450 enzymes (CYP3A4 and CYP2D6), decreasing enzyme activity by 50% [45], as well as drug transporters (hOATP1A2 and hOATP2B1), with IC50 of 37.3 ± 1.3 and 2.0 ± 1.7 μL, respectively [46]. In humans, pinocembrin is mainly metabolized into sulfonated and glucuronidated conjugates. These properties highlight the need to consider potential drug–drug interactions during its clinical application (Table 1) [47].

## 6. Excretion

Alpinetin glucuronidation occurred during phase II metabolism using pooled human liver microsomes (pHLMs), pooled human intestine microsomes (pHIMs), and recombinant UDP-glucuronosyltransferases (UGTs). This process generated a single major glucuronide metabolite, which was subsequently excreted via transporter-mediated pathways. UGT1A3 enzyme exhibited the highest catalytic activity toward alpinetin (CLint = 66.5 μL/min/nmol), followed by UGT1A1 (48.6 μL/min/nmol), UGT1A9 (21.0 μL/min/nmol), UGT2B15 (16.7 μL/min/nmol), and UGT1A10 (1.60 μL/min/nmol). Correlation analyses with established activity markers confirmed the major contributions of UGT1A1, UGT1A3, UGT1A9, and UGT2B15 in hepatic glucuronidation of alpinetin [48,49,50,51]. Similar to alpinetin, pinocembrin, or PCBG, underwent glucuronidation during phase II metabolism to produce UGT1A3, UGT1A1, UGT1A9, UGT2B15, and UGT1A10 enzymes. These compounds were mainly excreted through feces. The cumulative excretion rates of PCBG in rat urine, bile, and feces were 4.5 ± 2.4%, 0.2 ± 0.1%, and 18.4 ± 10.5%, respectively (Table 1) [52,53,54].

## 7. Pharmacodynamics of Alpinetin and Pinocembrin

Pharmacodynamics refers to the molecular, biochemical, and physiological effects of alpinetin and pinocembrin. It describes the function of target molecules through receptor binding, post-receptor signaling, and chemical interactions. They stimulate the activation of receptors through downstream pathways, which inhibit receptors, antagonize or block them, or stabilize them, or act via direct chemical interactions. These influence receptor activity without classical agonism or antagonism [55].

Thus, alpinetin and pinocembrin exert broad pharmacodynamic effects on inflammatory and metabolic pathways. Alpinetin suppresses pro-inflammatory cytokine signaling and promotes apoptosis in tumor cells [56,57]. However, pinocembrin has cardioprotective and hepatoprotective effects through antioxidant activity and regulation of mitochondrial function [58,59]. They are plant-derived bioactives. This integrates receptor-mediated signaling with broader biochemical modulation, producing anti-inflammatory, anti-tumor, and organ-protective outcomes with the regulation of immune and metabolic homeostasis.

## 8. Anti-Inflammatory Activity

Inflammation is a response to harmful stimuli, which has defensive effects but may also lead to many inflammatory diseases or even death. A great deal of scientific research has proved that alpinetin and pinocembrin have obvious anti-inflammatory effects on inflammation in the respiratory system, digestive system, reproductive system, and motor system.

### 8.1. Anti-Inflammatory Effects Against Respiratory Diseases

#### 8.1.1. Allergic Asthma

Allergic asthma is the most common type of asthma, and its pathological feature is airway inflammation. The inflammatory response driven by T helper 2 (Th2) cells, elevated IgE, and the NF-κB-related signal transduction pathway are important aspects in the pathogenesis of allergic asthma [60]. NF-κB is the key factor and plays a significant regulatory role in the inflammatory response [61]. Heme oxygenase (HO), the rate-limiting enzyme in heme catabolism, is also involved; HO-1, induced under oxidative stress, is regulated by the PI3K/Akt/NF-κB pathway and contributes to sustaining the inflammatory response [62]. Alpinetin has been reported to attenuate inflammatory responses in OVA-induced allergic asthma by modulating the PI3K/AKT/NF-κB and HO-1 signaling pathways, as well as inhibiting the levels of serum IgE and IL-4 in mice [63], whereas pinocembrin significantly reduced the Th2 cytokines interleukin (IL)-4, IL-5, and IL-13 in BALF and OVA-specific IgE in serum to suppress the phosphorylation of inhibitor-κBα (IκBα) and NF-κB subunit p65 activation in lung tissue of OVA-sensitized mice [64].

#### 8.1.2. Chronic Obstructive Pulmonary Disease (COPD)

COPD is a common chronic disease characterized by continuous airflow restriction. It includes chronic bronchitis and/or emphysema, which may further develop into cor pulmonale and respiratory failure, with a very high incidence, disability, and mortality. The pathogenesis depends on an abnormal inflammatory reaction to toxic gases or particles [65]. Previous studies have shown that alpinetin inhibited alveolar cell apoptosis and fibrosis induced by inflammation by downregulating the expressions of caspase-3, caspase-9, TGF-β1, TNF-α, IL-6, and α-SMA [66]. Pinocembrin inhibited lipopolysaccharide (LPS)-stimulated inflammatory responses in macrophages, which regulated the TLR4-NF-κB signaling pathway and suppressed the activation and assembly of NLRP3 inflammasomes [67]. 

Bronchodilators, like anticholinergic drugs, beta 2 receptor agonists, and theophylline, are the major drugs used to treat COPD, but these drugs have adverse reactions, highlighting the need to develop new bronchodilators. The main subtype of bronchial muscarinic receptor is M3 (CHRM3), and this exhibits potent bronchodilator effects through the inhibition of CHRM3. Alpinetin possesses significant bronchodilator effects, stably binding to CHRM3, which not only inhibits CHRM3 expression but also suppresses acetylcholine (ACh) [68]. Different from alpinetin, pinocembrin has been shown to reduce total cells, the DNA amount, and IL-1, IL-6, IL-8, TNF-α, and TGF levels in bronchoalveolar lavage fluid (BALF), as well as NF-κB levels. This ameliorates mycoplasma pneumoniae-provoked inflammation and oxidative stress. It may act as a therapeutic agent for the treatment of pneumonia in the future [69].

Idiopathic pulmonary fibrosis (IPF) is a chronic and fatal respiratory disease similar to COPD. Pulmonary fibrosis is also a typical pathological change in COPD. Alpinetin alleviated pulmonary fibrosis by inhibiting fibroblast differentiation and proliferation and promoting cell apoptosis via the TGF-β/ALK5/Smads pathway [70]. Alpinetin also exhibited a protective effect on LPS-induced lung epithelial cell injury by inhibiting p38 and ERK1/2 signaling via aquaporin-1 [71]. Pinocembrin was associated with significantly lower numbers of immunopositive CD8+ and CD4+ T cells in the lung parenchyma, as well as improved lung pathology and functional compliance in a sheep model of pulmonary fibrosis [72].

Alpinetin and pinocembrin have anti-inflammatory functions in respiratory disease models. The present study limits direct comparison, focusing only on their complementarity. Regarding the mechanism of respiration, NF-κB signaling is one of the general mechanisms of respiration to suppress airway inflammation. However, alpinetin modulates the PI3K/Akt and HO-1 pathways. Pinocembrin interacts with inflammasome assembly and Th2 cytokine suppression. These findings share a common anti-inflammatory axis and contribute to regulatory functions.

### 8.2. Anti-Inflammatory Effects Against Digestive Diseases

#### 8.2.1. Ulcerative Colitis

Ulcerative colitis is an important clinical subtype of inflammatory bowel disease. It is characterized by abdominal pain, recurrent diarrhea with pus/blood, and tenesmus. The pathogenesis of ulcerative colitis is not clear. It is generally considered to be the result of multiple factors such as environment, genetics, immunity, and intestinal microorganisms, in which changes in cytokine levels, damage to the intestinal epithelial barrier, and immune cells play a crucial role [73].

There are many cytokines involved in ulcerative colitis, among which the expression of pro-inflammatory factors IL-1, IL-6, IL-8, and TNF-α is significantly upregulated during its pathogenesis. In addition, the toll-like receptor 4 (TLR4)/NF-κB pathway is also closely related to it. Alpinetin reduced the levels of IL-6 and TNF-α and inhibited the activation of MAPK and NF-κB simultaneously in dextran sodium sulphate (DSS)-induced ulcerative colitis in mice [74]. Meanwhile, methyl CpG-binding protein 2 may promote the inhibitory effects of alpinetin on IL-6 and TNF-α in RAW246.7 mononuclear macrophages [75]. The mechanism of alpinetin inhibition of the expression of IL-6 was studied thoroughly from two aspects. Alpinetin promoted H3K9 deacetylation at the IL-6 promoter and reduced the content of intracellular transcription factor P40, which interfered with the synthesis of IL-6 [76]. It also regulated CpG dinucleotide methylation at the IL-6 promoter and reduced the transcription level of IL-6 by activating PPARs and DNA methyltransferase 3A (DNMT3A) [77]. TLR4, a member of the toll-like family, leads to an inflammatory response by activating the NF-κB signaling pathway. It has been reported that alpinetin reduced the levels of TNF-α and IL-1 β by reversing the upregulation of TLR4 induced by lipopolysaccharide (LPS) and inhibiting the NF-κB signaling pathway. In addition, alpinetin improved ulcerative colitis via the inhibition of nucleotide-binding oligomerization domain-like receptor family, pyrin domain-containing 3 (NLRP3) [78].

The function of the intestinal epithelial barrier is mainly regulated by tight junction at the top of epithelial cells. The integrity of tight junctions determines the function of the intestinal epithelial barrier. Injury of the intestinal epithelial barrier is considered to be a key pathophysiological process in response to gastrointestinal infection and inflammation and plays a critical role in gastrointestinal inflammatory responses [79]. Studies have shown that alpinetin improves intestinal barrier homeostasis in the following four ways: (a) restoring the integrity and permeability of the intestinal epithelial cell barrier by regulating the expression of tight junction proteins such as transmembrane proteins and peripheral membrane proteins [74]; (b) inhibiting the IL-6/STAT3 pathway and reducing colonic inflammatory responses [74]; (c) activating the Nrf2/HO pathway, which is a protective mechanism of antioxidant stress, and mediating intestinal epithelial barrier function in ulcerative colitis [80]; and (d) regulating the AHR/suv39h1/TSC2/mTORC1 signaling pathway, coordinating the relationship between autophagy and apoptosis to inhibit intestinal epithelial cell apoptosis and increase the production of autophagosomes to improve colitis [81].

Immune dysfunction of the intestinal mucosa is another important pathogenic factor in ulcerative colitis. The imbalance of T helper 17 cells (Th17)/regulatory T cells (Treg) is vital in ulcerative colitis. Aromatic hydrocarbon receptors are a kind of transcription factor that regulate the differentiation of a variety of T cells after being activated by ligands. It was proven that alpinetin not only increased the mRNA and protein expression of aromatase receptor target gene CYP1A1 in the mouse colon but also promoted the differentiation of Treg through some signal pathways to improve ulcerative colitis [82]. Alpinetin attenuated ulcerative colitis through anti-inflammatory effects, protecting the intestinal epithelial barrier and promoting regulatory T-cell differentiation.

However, pinocembrin eased the severity of dextran sulfate sodium (DSS)-induced colitis in mice by suppressing the abnormal activation of the TLR4/NF-κB signal pathway in vivo. It inhibited the binding of LPS to myeloid differentiation protein 2 (MD2), thereby blocking the formation of receptor multimer TLR4/MD2/LPS through the regulation of proteins like ZO-1, Claudin-1, Occludin, and JAM-A in the intestinal microbiota, thereby contributing to the restoration of intestinal barrier integrity [83].

#### 8.2.2. Pancreatitis-Induced Lung Injury

Severe acute pancreatitis has a rapid onset, often accompanied by multiple organ injury, especially acute lung injury. Inflammation is a key step in acute lung injury induced by this condition. TNF-α is an important cytokine involved in the whole process [40]. Aquaporin is the main channel of rapid water transport, which maintains water balance in the process of glycerol metabolism. A decrease in aquaporin in lung tissue aggravates the degree of pancreatitis-related lung injury. Alpinetin reduced the levels of ICAM-1 and TNF-α, increased the expression of aquaporin, and promoted the proliferation of pulmonary microvascular endothelial cells, which alleviated the acute lung injury induced by severe acute pancreatitis through p38/ERK1/2 signaling modulation [41,42,43]. Conversely, pinocembrin downregulated miR-34a-5p expression and upregulated the protein levels of peroxisome proliferator-activated receptor alpha (PPAR-α) and Sirtuin 1 (SIRT1), as well as the gene expression level of the inhibitor protein of NF-κB (IκB-α), maintaining the Bax/Bcl-2 ratio in acute pancreatitis [84,85].

### 8.3. Anti-Inflammatory Effects Against Reproductive Diseases

#### 8.3.1. Mastitis

Mastitis is a common disease in women, and acute suppurative mastitis is the most common type. It often occurs during lactation and is called a “breast carbuncle” according to traditional Chinese medicine theory. The incidence rate in primiparas is quite high, from 2% to 4%. It brings great pain to pregnant women, which affects the health of infants because they cannot continue breastfeeding [86]. Traditional Chinese medicine is an early treatment method. The adhesion and infiltration of leukocytes, especially neutrophils, is an important feature of the acute inflammation response, and myeloperoxidase (MPO) is a key indicator of the degree of neutrophil aggregation and infiltration [87]. Some studies have investigated LPS-induced mouse mastitis models and primary mouse mammary epithelial cells to observe the effects of alpinetin. The results showed that alpinetin significantly inhibited the infiltration of neutrophils and the activation of MPO, reduced the levels of pro-inflammatory cytokines TNF-α, IL-1 β, and IL-6, and downregulated the phosphorylation of IκB-α as well as NF-κB p65 and TLR4 expression induced by LPS in vivo and in vitro. These molecular changes led to pathological changes, including reduced inflammatory cell infiltration in mammary tissue, decreased edema and tissue swelling, preservation of alveolar structure, and reduced necrosis and degeneration of epithelial cells, compared to untreated LPS-induced mastitis. Thus, alpinetin inhibits the TLR4/NF-κB signaling pathway to protect against mastitis and is expected to become a promising therapeutic agent [88]. Pinocembrin is a strong candidate for mastitis therapy, but experimental validation in bovine or lactational mastitis models requires further investigation.

#### 8.3.2. Endometritis

Endometritis refers to inflammation of the endometrium, which in severe cases may extend into the myometrium and progress to myositis. Additionally, incomplete resolution of acute inflammation often progresses to chronic endometritis, a major contributor to spontaneous abortion [89]. In murine models of LPS-induced endometritis, alpinetin administration suppressed inflammatory factor production and MPO activity, alleviated uterine histopathology, and upregulated PPAR-γ expression in a dose-dependent manner. These effects were associated with inhibition of NF-κB activation, highlighting the protective role of PPAR-γ signaling [90]. Although there is no direct study on pinocembrin in endometritis, its established pharmacological activities include inhibition of the NF-κB and MAPK pathways, suppression of pro-inflammatory cytokines IL-1β, TNF-α, and IL-6, and antioxidant properties for therapeutic purposes.

Alpinetin and pinocembrin have anti-inflammatory effects in LPS-induced mouse models of mastitis and endometritis. However, these findings remain preliminary. They rely only on mouse models, limiting extrapolation to human reproductive disease. Thus, the current evidence should be interpreted as indicative of their immunomodulatory potential rather than as established therapeutic efficacy.

### 8.4. Anti-Inflammatory Effects Against Locomotor Diseases

#### 8.4.1. Osteoarthritis

Osteoarthritis is a common chronic degenerative disease characterized by joint swelling, pain, deformity, and limited activity. The pathogenesis of osteoarthritis is multifactorial, involving pro-inflammatory cytokines such as TNF-α and interleukins, matrix metalloproteinase-13 (MMP-13), and activation of NF-κB signaling [91,92,93]. Alpinetin has been shown to reduce TNF-α-induced MMP-13 expression while upregulating B-cell lymphoma-2 (Bcl-2) and cyclin-dependent kinase 1 (CDK1). These effects protect chondrocytes from LPS-induced injury and attenuate osteoarthritis progression in mice, primarily through inhibition of NF-κB nuclear translocation [94,95,96]. In human chondrocytes, pinocembrin significantly inhibited TNF-α-induced phosphorylation and degradation of IκBα, thereby blocking NF-κB activation. This suppression reduced the expression of MMP-1, MMP-3, and MMP-13 at both mRNA and protein levels, suggesting a protective role against extracellular matrix degradation [97].

#### 8.4.2. Foot Inflammation

Carrageenan is a pro-inflammatory agent used in acute inflammation models. It induces acute inflammatory reactions such as swelling and tension pain in the foot based on diastolic local capillaries, increased permeability, and inflammatory factors following subendothelial injection into the sole [98]. It has been reported that alpinetin decreased the levels of MPO, TNF-α and IL-1, increased the expression of PPAR-γ, and inhibited the phosphorylation of NF-κB p65 in a dose-dependent manner in a mouse model of foot swelling induced by carrageenan. Consequently, it exerts anti-acute inflammation effects through the PPAR-γ/NF-κB signaling pathway [99]. Alpinetin has significant protective effects on inflammation in multiple organs and systems through various inflammatory signaling pathways. On the other hand, pinocembrin alleviated arthritis symptoms by markedly reducing joint erosion and the infiltration of inflammatory cells, an effect associated with modulation of the transcription factor SRY-related HMG-box 4 (Sox4). Dysregulation of the Sox4/Stat3 signaling axis was linked to altered expression of tumor necrosis factor-α, nuclear factor kappa B, and cyclooxygenase-2, as well as regulatory microRNAs including miR-132, miR-202-5p, and miR-7235. These findings suggested that pinocembrin exerts protective effects in arthritis through targeting Sox4/Stat3-mediated inflammatory pathways [100].

### 8.5. Anti-Inflammatory Effects Against Cardiovascular Diseases

Acute myocardial infarction (AMI) is a prevalent cardiovascular disease. Inflammation, necrosis, and cardiac insufficiency, resulting from myocardial infarction, eventually lead to congestive heart failure. Regulating myocardial inflammation has become an important target in the treatment of AMI. Previous studies have shown that alpinetin selectively targeted TLR4/MyD88/NF-κB signaling and significantly attenuated key AMI pathologies, including inflammatory infiltration, CD68+ macrophage activation, IL-6/TNF-α release, collagen deposition, and cardiomyocyte apoptosis [101]. Pinocembrin ameliorated cardiac function and attenuated remodeling in PIHF by scavenging reactive oxygen species and activating the Nrf2/HO-1 signaling pathway. These effects were accompanied by upregulation of nuclear factor erythroid 2-related factor 2 (Nrf2) and heme oxygenase-1 (HO-1), thereby reducing oxidative stress and improving outcomes in heart failure [102].

## 9. Anti-Tumor Effects

According to the latest data from the WHO, the number of cancer patients in 2020 reached 19.3 million, and the number of deaths increased to 10 million [103]. Breakthroughs have been made in the field of tumor research, such as Chimeric Antigen Receptor T-cell Immunotherapy (Car-T), programmed death-1 (PD-1), and its ligand PDL-1 [104]. However, there are still many difficulties in the treatment of tumors, including individual differences in the efficacy of anti-tumor drugs and side effects. The development of suitable anti-tumor drugs, particularly those derived from herbal medicines, remains a critical priority. However, the evidence for alpinetin and pinocembrin is limited to cytotoxicity studies in cell line models, and translation to clinical efficacy requires further investigation.

### 9.1. Gastrointestinal Cancer

#### 9.1.1. Gastric Cancer

Gastric cancer is one of the most common gastrointestinal cancers, and it tends to occur at younger ages. However, the incidence rate and mortality rate in China are decreasing, and males aged 60~69 years are a high-risk group for gastric cancer according to the findings of the China National Cancer Center [105]. Alpinetin inhibited proliferation and induced apoptosis of gastric cancer cells in a dose-dependent and time-dependent manner. The mitochondrial-dependent endogenous apoptosis pathway may be activated by the translocation of Bcl-2-associated X protein (Bax). Alpinetin promoted the translocation of mitochondrial Bax and Bcl-2 in the early stage of apoptosis, which resulted in a decrease in mitochondrial membrane potential and the release of cytochrome C, and activated the caspase family to induce apoptosis of gastric cancer cells [106,107]. Pinocembrin suppressed the proliferation, migration, and invasion of gastric cancer cells through the regulation of miR-34a-5p expression. This modulation led to reduced levels of MMP2, MMP9, phosphorylated PI3K, and phosphorylated AKT proteins, accompanied by an increased proportion of cells in the G0/G1 phase and a corresponding decrease in the S-phase population [108].

#### 9.1.2. Pancreatic Cancer

Pancreatic cancer is a malignant tumor in the digestive tract. Its incidence rate and mortality rate have increased significantly in recent years. This cancer is highly malignant and known as the “king of cancer”. The survival rate of pancreatic cancer is about 10%, making it one of the malignant tumors with the worst prognosis, due to obscure and atypical clinical symptoms, a low early diagnosis rate, high operative mortality, and a poor recovery rate [109]. Pancreatic cancer cell lines were treated with different doses of alpinetin at different times to observe its effects on cell growth, apoptosis, and the cell cycle. The results indicated that the proliferation of three kinds of pancreatic cancer cell lines was inhibited, and the apoptosis of BxPC-3 cells was induced in a dose- and time-dependent manner. Its mechanism may be related to regulating the expression of the cell survival-promoting factor Bcl-2 family and apoptosis inhibitor XIAP, releasing cytochrome C, and activating apoptosis protein caspases [110]. Pinocembrin inhibited the migration of Panc-1 cells by regulating the epithelial–mesenchymal transition (EMT) and induced cell cycle arrest at the G2/M phase, thereby contributing to its anti-proliferative effect. These findings suggest that pinocembrin may serve as a potential alternative therapeutic agent for pancreatic cancer [111].

### 9.2. Gynecological Tumors

#### 9.2.1. Ovarian Cancer

Ovarian cancer is one of the most common malignant tumors in female genital organs, and its incidence rate is second only to cervical cancer and uterine cancer. The mortality rate of epithelial ovarian cancer is the highest of all kinds of gynecologic tumors. The STAT3 signaling pathway is an important pathway for a variety of malignant tumors. STAT3 phosphorylation participates in the occurrence, proliferation, invasion, drug resistance, and recurrence of ovarian cancer by promoting the expression of downstream protooncogene c-myc, anti-apoptotic protein survivin, Mcl-1, and cyclin D1 [112]. Studies have shown that alpinetin inhibited the proliferation of ovarian cancer cells in a dose-dependent manner, reduced the diameter of tumor microspheres, and prevented tumor cell metastasis. In addition, the effects of alpinetin on ovarian cancer were attributed to downregulated p-STAT3, increased pro-apoptosis proteins, and decreased anti-apoptotic proteins [113,114]. Pinocembrin exerted anti-tumor effects in ovarian cancer cells primarily through regulation of epithelial–mesenchymal transition (EMT)-associated cadherin expression and modulation of GABAB receptor signaling, leading to reduced proliferation, impaired migration, and enhanced apoptosis [115].

#### 9.2.2. Breast Cancer

Breast cancer is the most common cancer in women worldwide, accounting for 11.7% of diagnosed cancer cases. Alpinetin has been applied for significant breast cancer regression [116], inhibiting the production of ROS in mitochondria, the activation of NF-κB, and the transcription of hypoxia-inducible factor 1-α (HIF-1α) in breast cancer cells in a dose-dependent manner. Consequently, inhibition of the ROS/NF-κB/HIF-1α signal pathway contributed to the anti-breast cancer effect of alpinetin. Pinocembrin exhibited anti-proliferative activity in breast cancer cells by inducing G2/M-phase arrest and promoting apoptosis. These effects were associated with downregulation of cell cycle- and apoptosis-related proteins, including cyclin B1, Cdc2, PARP1, Bcl-2, and survivin, alongside upregulation of cleaved PARP1, cleaved caspase-3, cleaved caspase-9, and BAX. Mechanistically, these changes were mediated through inhibition of the PI3K/AKT signaling pathway [117].

## 10. Cardiovascular Protection

### 10.1. Anti-Apoptotic Effects in Cardiomyocytes

The incidence rate of cardiovascular diseases has always been the highest in China, among which acute myocardial infarction (AMI) is the most common. Apoptosis is a critical mechanism in AMI. Apoptosis induces cardiac insufficiency and exacerbates myocardial ischemia, hypoxia, and reperfusion injury in the early stage and after AMI, respectively [118]. A cardiomyocyte apoptosis model induced by neonatal rat cardiomyocyte serum deprivation [56] showed that alpinetin inhibited cardiomyocyte apoptosis in a concentration-dependent manner, which was blocked by δ Opioid receptor antagonists, PKC inhibitors, and ERK inhibitors. Alpinetin may have maintained the stability of the mitochondrial membrane potential of damaged cardiomyocytes, inhibited the release of cytochrome C, and prevented the translocation of Bax from the cytoplasm to mitochondria. This study suggested that alpinetin induced endogenous protection of cardiomyocytes through the PKC/ERK signaling pathway and δ receptor. In addition, alpinetin reduced the apoptosis rate of cardiomyocytes in a concentration-dependent manner and downregulated the expression of apoptotic proteins such as caspase-3 and Bax [119]. This has a good effect on cardiomyocyte apoptosis. Pinocembrin inhibited the RhoA/ROCK signaling pathway, an effect potentially linked to its anti-atrial fibrillation activity through the suppression of apoptosis, which reduced the protein expression of Cav1.2, Kv4.2, Kv4.3, and connexin 40 (CX40) in right atrial tissue, thereby contributing to the prevention of pulmonary arterial hypertension [120].

### 10.2. Vasodilatation

Vascular endothelial dysfunction is characterized by a decrease in or the disappearance of endothelium-dependent vasodilatation. It is a pathological change in many cardiovascular diseases, such as atherosclerosis, hypertension, and diabetes. This is a central process in their pathogenesis. Vascular endothelial dysfunction is related to a decrease in NO activity [121,122]. NO, synthesized by endothelial cells via nitric oxide synthase (NOS), is a corresponding endothelium-derived vasodilator. To regulate vascular tension, it also has a variety of cardiovascular-protective effects, for example, anti-oxidation, inhibiting platelet aggregation, and the proliferation of vascular smooth muscle cells [123].

Alpinetin has been applied to induce the relaxation of the mesenteric artery of rats precontracted by phenylephrine, and the IC 50 was 27.5 μmol·L^−1^. The vasodilator effects were blocked by NOS inhibitor N-nitro-L-arginine methyl ester (L-NAME), methylene blue, and endothelial removal. On the contrary, L-arginine (NO precursor) recovered its vasodilation. Alpinetin also inhibited extracellular Ca^2+^ influx and intracellular Ca^2+^ release. These results suggested that alpinetin exerted vasodilatory effects through NO-mediated endothelium-dependent relaxation and endothelium-independent relaxation, like regulation of cytoplasmic calcium concentration and PKC-dependent contraction [124]. Pinocembrin exerted cardioprotective effects in chronic ischemic heart failure by attenuating autonomic nerve remodeling and reducing susceptibility to ventricular arrhythmias. Mechanistically, pinocembrin upregulated the expression of ion channel proteins Cav1.2 and Kv4.3, thereby ameliorating the shortening of the action potential duration (APD) and decreasing both the incidence and duration of ventricular fibrillation (VF). In parallel, it suppressed nerve growth factor (NGF) expression, contributing to improved autonomic regulation. Structural benefits were also observed, as pinocembrin reduced infarct size and myocardial fibrosis, accompanied by enhanced connexin 43 (CX43) expression, which supports gap junction integrity and electrical conduction. Collectively, these findings indicate that pinocembrin mitigates pathological remodeling at both the electrophysiological and structural levels, thereby conferring protection against arrhythmogenesis in chronic ischemic heart failure [125].

### 10.3. Other Cardiovascular-Protective Effects

Inflammation and pro-inflammatory factors are of great significance in the pathogenesis of cardiovascular diseases. IL-6 and TNF-α are the most studied inflammatory factors and are involved in the occurrence of coronary atherosclerosis and plaque formation [126,127]. IL-6 is the main participant in the acute phase of the coronary inflammatory response. It induces the liver to produce acute inflammatory proteins such as C-reactive protein, which aggravates inflammation and triggers chain amplification. It can also promote platelet aggregation and coronary artery smooth muscle cell proliferation to form plaque and further act on T lymphocytes in the plaque to secrete interferon, leading to plaque rupture [128,129]. IL-6 is associated with increased collagen production in myocardial fibroblasts [130]. This may prolong the elevation of TNF-α, which promotes cardiac remodeling and dysfunction after myocardial infarction [131].

Lungkaphin et al. demonstrated that pinocembrin attenuates cardiac arrhythmia and reduces infarct size in the context of acute myocardial ischemia/reperfusion (I/R). Administration of pinocembrin conferred significant cardioprotective effects, as evidenced by improved cardiac function, decreased arrhythmic events, and reduced infarct area. These protective actions were attributed to its anti-apoptotic and anti-oxidative properties, together with its capacity to enhance connexin 43 (Cx43) phosphorylation in the ischemic myocardium, thereby supporting gap junctional communication and myocardial electrical stability [132].

Our latest study investigated alpinetin, which protected against acute myocardial infarction in rats by inhibiting the activation of the inflammatory signaling pathway and downregulating the expressions of cytokines. Significantly, the antithrombotic [133] and antioxidant effects [134] of alpinetin may also contribute to its cardiovascular protection.

## 11. Liver and Kidney Protection

### 11.1. Non-Alcoholic Fatty Liver Disease

Non-alcoholic fatty liver disease (NAFLD) is a clinicopathological syndrome characterized by excessive fat deposition in hepatocytes, excluding alcohol and other clear factors. It is an acquired metabolic stress-induced liver injury, including simple fatty liver, non-alcoholic steatohepatitis, and liver cirrhosis. It is closely related to insulin resistance and genetic susceptibility. Non-alcoholic fatty liver disease directly leads to cirrhosis, for which the incidence within 10 years is as high as 25%, and hepatocellular carcinoma [135]. Furthermore, it participates in the pathogenesis of cardiovascular and cerebrovascular diseases such as type 2c diabetes and atherosclerosis [136]. With the global epidemic trend of obesity and its related metabolic syndrome, NAFLD has become an important cause of chronic liver disease and a public health problem seriously threatening people’s lives, especially in developed countries and rich areas of developing countries [137]. In an NAFLD model of high-fat fed mice, it was identified that alpinetin alleviated lipid accumulation, abnormal lipid metabolism, inflammation, and oxidative stress induced by a high-fat diet in the liver. Its antioxidant and anti-inflammatory effects were involved in activating the SOD1/Nrf-2/HO-1 pathway, reducing the level of thioredoxin interacting protein (TXNIP)/xanthine oxidase (XO), and inhibiting theTLR4/NF-κB signaling pathway, respectively [138]. Pinocembrin ameliorated glucose and lipid metabolic disturbances, inflammation, and oxidative stress in non-alcoholic fatty liver disease (NAFLD) through activation of the Nrf2/HO-1 signaling pathway and suppression of NF-κB activity. High-fat diet (HFD) feeding markedly reduced the expression of Nrf2 and HO-1; however, pinocembrin treatment restored their levels and promoted enhanced nuclear translocation of Nrf2, thereby reinforcing antioxidant defense and attenuating hepatic injury [139].

### 11.2. Liver and Kidney Injury

Inflammation is one of the important mechanisms of the immune response, which has always been a focus of life science research. It is closely related to many diseases such as tumors, cardiovascular and cerebrovascular diseases, autoimmune diseases, and neurodegenerative diseases. Lipopolysaccharide (LPS) is the standard reagent for establishing an acute inflammation model due to its easy control, good model reproducibility, and easy identification and detection of its effects on organisms. The effects and mechanisms of alpinetin on acute renal injury induced by LPS in mice have been studied [99], and the results showed that alpinetin alleviated renal histopathological changes, decreased the levels of blood urea nitrogen and creatinine, and inhibited the production of ROS, malondialdehyde (MDA), and inflammatory cytokines such as TNF-α, IL-6, and IL-1β in renal tissue. Mechanistically, alpinetin not only upregulated the expression of Nrf2 and HO-1 but also inhibited the expression of TLR4 and NF-κB in a dose-dependent manner. This indicates antioxidation and anti-inflammatory effects on LPS-induced renal injury through activation of the Nrf2/HO-1 pathway and inhibition of the TLR4/NF-κB pathway, respectively. Subsequently, some scholars studied a mouse liver injury model induced by LPS/D-galactosamine (D-gal). Alpinetin was shown to improve the infiltration of hepatic inflammatory cells, restore the structure of hepatic lobules, and inhibit the activity of hepatic myeloperoxidase along with the levels of MDA, TNF-α, and IL-1β in a dose-dependent manner. The mechanism involved in this was similar to that described above [98]. The latest research indicated that alpinetin protected against liver fibrosis through anti-inflammatory, antioxidant, and anti-angiogenesis effects [101].

The anti-inflammatory activity of pinocembrin was evidenced by its inhibition of key inflammatory effectors, p-JNK and NF-κB p65, thereby disrupting TNF-α and IL-6 downstream signaling in cisplatin-induced liver injury. In addition, pinocembrin suppressed hepatic apoptotic signaling by reducing caspase-3 activation and normalizing the Bax/Bcl-2 ratio. Through its combined actions on oxidative stress, the TAK1–inflammatory cascade crosstalk, and apoptosis, pinocembrin conferred significant hepatoprotective effects against cisplatin-induced hepatotoxicity [140]. Pinocembrin significantly attenuated LPS-induced injury in HK-2 cells by modulating endoplasmic reticulum stress, which in turn suppressed inflammatory responses, oxidative stress, and apoptosis. Moreover, pinocembrin reduced cytokine expression in a concentration-dependent manner. Collectively, these findings indicate that pinocembrin may serve as a promising therapeutic candidate for septic acute kidney injury [141].

### 11.3. Hepatic Ischemia–Reperfusion Injury

Ischemia–reperfusion (IR) injury is the phenomenon in which ischemic injury is further aggravated after the recovery of blood perfusion in ischemic organs. It can occur in various tissues and organs. Liver injury caused by IR is the main problem in liver transplantation and resection [142]. In a liver IR animal model and hypoxia/reoxygenation hepatocyte model, researchers observed that alpinetin not only improved the inflammatory responses and apoptosis induced by IR but also inhibited the activation of the NF-κB/MAPK pathway in hepatocytes after hypoxia/reoxygenation. These results suggested that alpinetin may be a promising drug for the treatment of hepatic IR injury [143].

Pinocembrin conferred significant protection against hepatic ischemia–reperfusion injury by inhibiting the HMGB1/TLR4 signaling pathway. This intervention attenuated hepatocyte apoptosis and suppressed the expression of HMGB1 and TLR4. Furthermore, pinocembrin reduced reactive oxygen species levels, mitochondrial membrane potential, apoptotic cell counts, and Bcl-2 protein expression while concomitantly enhancing Bax protein expression. Collectively, these findings highlight the hepatoprotective potential of pinocembrin through coordinated regulation of oxidative stress, apoptosis, and HMGB1/TLR4-mediated inflammatory signaling [144].

### 11.4. Other Effects

Alpinetin has inhibitory effects on a variety of microorganisms; for instance, the minimum inhibitory concentration against Helicobacter pylori was 1.25 μg·mL^−1^, indicating strong antibacterial activity compared with the positive drug metronidazole. It has certain antibacterial effects on *Staphylococcus aureus*, *Staphylococcus epidermidis*, *Escherichia coli*, etc. The minimum inhibitory concentrations are 1.275~2.550 mg·mL^−1^ and 1.925~3.850 mg·mL^−1^ respectively [145]. Alpinetin may have a protective effect on the nervous system, not only inhibiting neuroinflammation and neuronal apoptosis through the JAK2/STAT3 signaling pathway but also inhibiting mitochondrial inflammation and reducing aging-related cognitive deficits through the Drp1/HK1/NLRP3 pathway [146,147].

Pinocembrin inhibited the growth of aeromonas hydrophila by increasing cell membrane permeability and disrupting protein and DNA metabolism. The minimal inhibitory concentration (MIC) and minimum bactericidal concentration (MBC) were determined to be 256 μg/mL and 512 μg/mL, respectively. Treatment with pinocembrin significantly reduced lactate dehydrogenase activity and soluble protein content, while electrical conductivity and DNA exosmosis levels increased by 4 [148].

## 12. Discussion

Based on the above review information, there are some considerations regarding the therapeutic potential of “alpinetin” and “pinocembrin” in human diseases, including: (a) chemical structural effects; (b) different mechanistic actions; (c) a lack of clinical studies and limitations; (d) the application of nanotechnology; (e) regulatory challenges and pathways for botanical drugs; (f) current limitations; and (g) future directions and translational potential.

**Figure 4:**
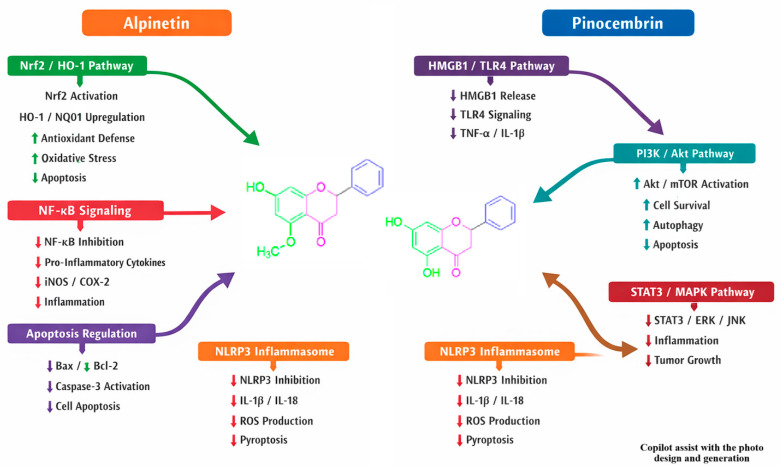
Molecular targets of alpinetin and pinocembrin.

(a) Alpinetin and pinocembrin share a flavanone backbone, but alpinetin’s methoxy substitution increases lipophilicity, reducing its aqueous solubility from 21 ± 0.64% to 15.98 ± 1.93 mg/L. This enhanced lipophilicity facilitates passive membrane diffusion and stabilizes hydrophobic interactions. Alpinetin shows strong binding to human serum albumin (HAS) [149] and activation of PPAR-γ. In contrast, pinocembrin has a hydroxyl-rich structure that supports redox cycling and electrophilic interactions for its activation of the Nrf2 pathway. Glycosylation of pinocembrin increases polarity, limiting passive diffusion and necessitating enzymatic hydrolysis before absorption. Thus, methoxylation of alpinetin versus hydroxylation/glycosylation of pinocembrin results in different pharmacokinetic and pharmacodynamic profiles, linking SAR directly to the mechanism of action.

(b) Besides structural and binding variations, alpinetin and pinocembrin have different mechanistic actions and molecular targets (Table 2 and Figure 4). Alpinetin exerts its therapeutic potential mainly by modulating multiple signaling pathways, such as NF-κB/MAPK, PI3K/Akt, and PPAR-γ, as well as influencing cytochrome P450 enzymes and undergoing glucuronidation. These mechanisms allow it to confer anti-inflammatory, anti-tumor, cardiovascular protection, liver or kidney protection, and antibacterial properties [49]. In contrast, pinocembrin exerts its therapeutic potential primarily because it modulates multiple signaling pathways and cellular processes rather than acting on a single receptor target [28]. This pleiotropic activity includes inhibition of HMGB1/TLR4 signaling, regulation of endoplasmic reticulum stress, activation of Nrf2/HO-1 antioxidant pathways, suppression of NF-κB-mediated inflammation, and modulation of drug transporters and cytochrome P450 enzymes, providing broad protective effects against cardiovascular ischemia–reperfusion injury, inflammatory diseases, and bacterial infections.

For the comparative application of alpinetin and pinocembrin, they are structurally related flavonoids, which share overlapping pharmacological profiles, and the distinct pharmacokinetic (PK) or pharmacodynamic (PD) properties suggest different therapeutic niches. Preclinical evidence provides a foundation for anticipating how each compound may be preferentially applied in specific disease contexts. From a pharmacokinetic perspective, alpinetin demonstrates higher systemic exposure and slower clearance compared to pinocembrin. In rat models, alpinetin reached a C_max_ of approximately 386 ng/mL with an AUC of ~912 ng/mL·h, whereas pinocembrin achieved a Cmax of ~109 ng/mL and an AUC of ~138 ng/mL·h. These findings indicate that alpinetin possesses stronger bioavailability and more sustained systemic activity. Its clearance is primarily mediated through bile and urine, reflecting extensive glucuronidation and transporter-dependent excretion. Pinocembrin, in contrast, exhibits a longer half-life (2.5 h versus 1.6 h for alpinetin) and predominant fecal excretion, suggesting slower elimination but lower systemic exposure overall. These PK differences imply that alpinetin may be more effective in acute inflammatory conditions requiring rapid and sustained suppression of cytokines, while pinocembrin may be better suited for chronic oxidative stress states where prolonged antioxidant activity is beneficial.

Pharmacodynamically, alpinetin exerts its effects mainly through modulation of the NF-κB/MAPK and PI3K/Akt signaling pathways, resulting in a 2–3-fold reduction in pro-inflammatory cytokines such as TNF-α, IL-6, and IL-1β. This mechanistic profile supports its preferential use in inflammation-driven disorders, including allergic asthma, ulcerative colitis, and tumor models where cytokine suppression and apoptosis induction are critical. Alpinetin’s ability to interact with muscarinic receptors (CHRM3) suggests potential utility as a novel bronchodilator in chronic obstructive pulmonary disease (COPD), complementing its anti-fibrotic effects in pulmonary fibrosis. Pinocembrin activates Nrf2/HO-1 signaling and inhibits HMGB1/TLR4 pathways, leading to an approximate 1.8-fold increase in antioxidant enzyme activity. This positions pinocembrin as a candidate for conditions characterized by oxidative stress and mitochondrial dysfunction, such as ischemic stroke, cardiomyopathy, and hepatic injury. Its regulation of endoplasmic reticulum stress and inflammasome assembly further underscores its potential in protecting organs from oxidative and inflammatory insults. In respiratory disease models, pinocembrin has shown efficacy in reducing Th2 cytokines and suppressing NF-κB activation, highlighting its dual anti-inflammatory and antioxidant roles.

Based on this information, alpinetin may be prioritized in hyper-inflammatory contexts where rapid cytokine suppression is essential, while pinocembrin may be favored in oxidative stress-mediated organ injury requiring sustained antioxidant defense. These complementary profiles suggest that the two flavonoids could even be explored in combination strategies, leveraging their distinct mechanisms to achieve broader therapeutic coverage.

(c) Although there is much research on cell and animal experiments on alpinetin and pinocembrin, there is a lack of clinical studies on humans. The only clinical study identified was a phase II trial of pinocembrin injection for ischemic stroke in Chinese patients, designed as a randomized, double-blind, placebo-controlled multicenter study. However, this trial was suspended without published safety or efficacy results. The registry lists its status as suspended since 06/2016, with no official reason disclosed by the sponsor. Typically, dosing was 10 to 30 mg per day intravenously for 5 to 14 days, and the treatment duration was 14 days with follow-up to day 90 for neurological assessment, according to ClinicalTrials.gov [150]. Therefore, there exists a research gap between animal experiments (preclinical) and clinical trials. Clinical studies on alpinetin and pinocembrin remain limited, primarily because of their “low oral bioavailability”, approximately 15.1% and <10% [54,149] for alpinetin and pinocembrin, respectively. Current evidence relies on in vitro experiments and animal models to demonstrate their anti-inflammatory and anticancer activities. As a result, robust clinical data in humans are insufficient. In future studies on alpinetin and pinocembrin, it may be possible to use nanotechnology to further improve their bioavailability and therapeutic efficacy, facilitating clinical translation.

To make the physicochemical and pharmacokinetic parameters of alpinetin and pinocembrin more critical, a data-driven evaluation should be used to normalize pharmacokinetic data across studies to allow comparison, which can apply drug-likeness assessment, such as Lipinski’s Rule of Five, to evaluate oral bioavailability through molecular weight, hydrogen bonding, and lipophilicity. A compound or drug is orally active if it fulfills the following requirements: molecular weight ≤ 500 Da, LogP ≤ 5 (lipophilicity), ≤5 hydrogen bond donors, and ≤10 hydrogen bond acceptors [151].

Alpinetin and pinocembrin have molecular weights under 300 Da; their hydroxyl and methoxy groups contribute to hydrogen bonding, and their lipophilicity (LogP values) is moderate, supporting membrane permeability. Alpinetin’s LogP is around 2.3–2.6 [49], and pinocembrin’s LogP is around 2.4–2.7 [152]. These properties indicate that alpinetin and pinocembrin are orally bioavailable candidates. However, systematic clinical evaluation is still required.

(d) Alpinetin demonstrates improved lipophilicity with its inherent methoxy substitution, and further structural modifications such as fluorination or glycosylation may stabilize metabolism and enhance absorption. In contrast, pinocembrin lacks methoxy groups, rendering it more susceptible to conjugation; methoxylation and prodrug esterification are promising strategies to improve its bioavailability. Beyond chemical modification, nanotechnology-based formulations have shown potential.

For example, Wei reported that alpinetin nanoparticles alleviate optic nerve injury induced by acute glaucoma via LRP1-PPARγ-mediated regulation of microglial lipid metabolism. The study demonstrated that AlpNPs exhibited efficient microglial uptake and sustained release, leading to reduced intracellular lipid accumulation, enhanced M2 polarization, and suppression of microglial proliferation and migration. Mechanistically, AlpNPs directly bound to LRP1 and strengthened its interaction with PPARγ, thereby activating the downstream LXRα-ABCA1 pathway, which plays a pivotal role in cholesterol efflux and anti-inflammatory responses [153]. Shen et al. reported pinocembrin-loaded polyethylene glycol succinate–vitamin E-modified liposomes with enhanced bioavailability and antihyperglycemic activity. The oral bioavailability of pinocembrin liposomes was enhanced by approximately 1.96-fold compared to free pinocembrin. Pharmacokinetic analysis revealed a Cmax of 1.700 ± 0.139 µg·mL^−1^, a mean residence time (MRT_0_–t) of 12.695 ± 1.647 h, and a half-life (T_1_/_2_) of 14.244 h. In diabetic mice, treatment with pinocembrin liposomes significantly reduced serum biomarkers, with decreases of 28.28% in aspartate aminotransferase (AST), 17.23% in alanine aminotransferase (ALT), 17.77% in interleukin-1 (IL-1), and 8.08% in tumor necrosis factor-α (TNF-α) [154]. This suggests potential utility in clinical settings for liver protection in diabetes. However, nanotechnology-based formulations for alpinetin and pinocembrin remain uncertain. The long-term toxicity and biocompatibility of carriers such as liposomes or polymeric nanoparticles have not been fully evaluated. Future work should focus on systematic safety evaluations, including chronic toxicity, biodistribution, and immunogenicity studies, alongside regulatory compliance assessments. Parallel strategies including chemical modifications such as methoxylation, fluorination, glycosylation, and prodrug esterification may offer an alternative pathway to enhance bioavailability without carrier-related risks. Recent studies on feasibility, safety, and regulatory compliance are essential before these nano-formulations advance toward clinical application.

(e) To date, neither alpinetin nor pinocembrin has been registered with the U.S. Food and Drug Administration (FDA), and they are not recognized as regulated botanical drugs for the treatment of human diseases according to the FDA’s Global Substance Registration System (GSRS) [155,156]. To achieve registration, several critical criteria must be satisfied, including rigorous safety evaluation [157], which depends on the purity and dosage of the natural product, as well as its regulatory classification as either a dietary supplement or a botanical drug. Dietary supplements do not require pre-market approval, whereas botanical drugs must undergo comprehensive phase I–III clinical trials to establish safety and efficacy (IND submission, phase I–III trials, NDA approval) [158]. Clinical investigations of alpinetin and pinocembrin remain scarce, with studies limited to early-stage trials (phase I or II), as noted above (c). This underscores the need for well-designed clinical studies to bridge the gap between promising preclinical findings and regulatory approval. Alpinetin and pinocembrin exhibit a wide range of pharmacological activities, but the clinical process is still ongoing for their use as anti-tumor, anti-inflammatory, cardiovascular protection, liver or kidney protection, and antibacterial drugs in humans.

(f) The limitations of the studies include reliance on in vitro assays and animal models. This restricts direct translation to human physiology. Many pharmacokinetic and pharmacodynamic results, such as CYP450 inhibition or cytokine modulation, are derived from rat microsomes or murine disease models. These systems differ significantly from human metabolism, immune responses, and drug–drug interactions. For example, alpinetin’s selective inhibition of CYP1A2 and pinocembrin’s transporter blockade have been observed, but these interactions in vivo remain uncertain. Similarly, anti-inflammatory and organ-protective effects have been demonstrated in OVA-induced asthma or LPS-stimulated macrophages, which only partially mimic complex human disease states. Variability in dosing regimens and extraction methods is another limitation. This complicates comparisons across studies and may exaggerate pharmacological effects. Genetic polymorphisms in human UGTs and CYP enzymes are not adequately addressed, limiting the understanding of inter-individual variability. These are preclinical findings, and there is an absence of robust clinical trials. Further validation is necessary before therapeutic application.

Meanwhile, there are differences in dosage, experimental models, and endpoints across the included studies, which limit the possibility of a robust quantitative comparison between alpinetin and pinocembrin, according to Table 2. However, alpinetin was usually evaluated at lower doses and with distinct mechanistic readouts, while pinocembrin studies employed higher doses and different endpoints, such as antioxidant enzyme activity. These methodological variations preclude direct normalization of data. Therefore, the comparative analysis should be interpreted as qualitative, highlighting complementary pharmacological actions and mechanistic diversity. These should be further standardized, and continuous studies under comparable conditions are essential to enable reliable quantitative evaluation and translational relevance.

(g) In future research, alpinetin and pinocembrin should be evaluated using standardized disease models to clarify their relative efficacy and mechanistic effects, including pharmacokinetic and pharmacodynamic profiling, for example, dose–response and long-term toxicology. This would establish a relationship between preclinical findings and clinical translation. Meanwhile, nanotechnology-based formulations have the benefit of low oral bioavailability, but their safety, biocompatibility, and regulatory compliance must be continuously investigated. Clinical trials designed with regulatory alignment should be conducted to fulfill the translational strategies for therapeutic effectiveness in human disease. The relative efficacy, pharmacological advantages, and translational relevance of alpinetin and pinocembrin are summarized in Table 3.

The structured quality assessment in Table 4, covering randomization, blinding, sample size, target, reproducibility, and funding, addresses limitations for evaluation in future investigations. Overall, alpinetin and pinocembrin studies carry a moderate-to-high risk of bias. These findings are preliminary, and future research should adopt standardized dosing, a clearly defined target, and rigorous reporting to strengthen translational relevance.

In summary, alpinetin and pinocembrin share structural similarity and overlapping pharmacological profiles. They have distinct mechanistic preferences that suggest complementary therapeutic potential. Alpinetin predominantly modulates NF-κB/MAPK and PI3K/Akt pathways, favoring anti-inflammatory and anti-tumor effects, whereas pinocembrin exerts stronger antioxidant and organ-protective actions through Nrf2/HO-1 and HMGB1/TLR4 signaling. Hence, alpinetin may be preferable for inflammation-driven disorders, whereas pinocembrin may be more suitable for oxidative stress-related conditions. However, there are some unknowns, confined to preclinical evidence. Future research should prioritize formulation innovation, including the use of nanotechnology to bridge the translational gap toward clinical validation and therapeutic application in humans.

To standardize pharmacokinetic profiling of alpinetin, Sprague–Dawley rats have been used to clarify its poor oral bioavailability, which is largely driven by extensive glucuronidation and efflux transporter activity [159]. Zhao et al. have consolidated alpinetin’s pharmacological actions under harmonized endpoints, reducing variability across models [49]. Meanwhile, parallel assays of pinocembrin in cardiovascular and cancer cell models have determined its antioxidant and cytoprotective effects side-by-side with alpinetin, which is a direct comparison of NF-κB suppression and Nrf2 activation [160]. Transporter-focused studies have highlighted pinocembrin’s inhibitory effects on hOATP1A2 and hOATP2B1 under standardized conditions, improving reproducibility across laboratories. These advances partially homogenize the dataset by aligning species, dosing regimens, and analytical methods while still acknowledging residual differences in metabolism and clearance. Incorporating these findings strengthens the translational framework and highlights the need for enantiomer-specific and transporter-focused studies to achieve full comparability in clinical development.

Currently, enantioseparation work has established standardized HPLC methods to quantify both alpinetin and pinocembrin enantiomers in *Alpiniae Katsumadai* semen, providing reproducible content values across batches [161]. Pinocembrin in Alzheimer’s disease models has demonstrated multi-target activity through network pharmacology, docking, and in vitro validation, linking antioxidant and neuroprotective effects to specific molecular targets under harmonized assays [162]. Additionally, the discovery of alpinetin and pinocembrin glucosides in *Penthorum chinense* offers structurally comparable derivatives that can be tested under unified pharmacological conditions [163]. These advances reduce reliance on narrative inference by aligning species, formulations, and analytical methods, thereby enabling more quantitative comparisons of pharmacological potency. Nonetheless, residual variability in metabolism and clearance persists, underscoring the need for enantiomer-specific PK–PD correlation and transporter-focused studies.

## 13. Conclusions

Flavonoids are the main active compounds isolated from traditional Chinese medicine. The present article highlights the therapeutic potential of the flavonoids alpinetin and pinocembrin, with diverse pharmacological activities, including anti-inflammatory, anti-tumor, cardioprotective, hepatoprotective, nephroprotective, and antibacterial functions.

However, current research on alpinetin and pinocembrin still has the following limitations: (1) Most of the studies are limited to cell and animal experiments, and there is not much evidence for clinical studies. (2) Some progress has been made on alpinetin and pinocembrin, as well as molecular targets in various diseases, but their signal transduction mechanisms and actions require further investigation (Table 2 and Table 3). (3) The pharmacological activities of flavonoids are diverse, and the effects of alpinetin and pinocembrin, especially in the protection of reproductive and cardiovascular systems, need to be expanded more deeply. (4) The use of nanotechnology may further improve the bioavailability of alpinetin and pinocembrin for therapeutic purposes and facilitate clinical translation. Overall, this review provides scientific evidence for alpinetin and pinocembrin, which are expected to have great significance for the development of anti-tumor, anti-inflammation, cardiovascular protection, liver or kidney protection, and antibacterial drugs. Alpinetin and pinocembrin show pharmacological activity in preclinical models, but clinical evidence is limited. This is necessary for the design of rigorous clinical trials, such as randomized, double-blind, placebo-controlled studies on inflammatory biomarkers, organ function, and patient-reported outcomes. Comprehensive toxicology data, particularly on long-term safety, immunogenicity of nano-carriers, and dose–response relationships in humans, are lacking. Nanotechnology formulations, standardized pharmacokinetic profiling, and regulatory-aligned toxicology assessments should be further investigated.

## 14. Future Aspects

Alpinetin and pinocembrin as therapeutic agents require systematic evaluation, including drug-likeness, bioavailability, toxicity, clinical relevance, and challenges in drug development. Drug-likeness involves physicochemical profiling, such as solubility, lipophilicity, LogP, molecular weight, and hydrogen bond donors/acceptors. Alpinetin shows higher systemic exposure (C_max_ ≈ 386 ng/mL, AUC ≈ 912 ng·h/mL) compared to pinocembrin (C_max_ ≈ 109 ng/mL, AUC ≈ 138 ng·h/mL). Alpinetin shows favorable systemic exposure and rapid clearance, and pinocembrin demonstrates a longer half-life, which may influence formulation strategies. Bioavailability remains a great challenge due to intestinal absorption variability and extensive glucuronidation, leading to <15% for alpinetin and <10% for pinocembrin, limiting oral efficacy. The design of nano-delivery systems, such as nanoparticles or prodrugs, can improve therapeutic outcomes. Toxicity and safety are concerns. Although preclinical studies report low toxicity, systematic dose-ranging and chronic toxicity studies in humans are lacking, and CYP450/transporter inhibition (CYP1A2, CYP3A4, CYP2D6, hOATP1A2/2B1) raises drug–drug interaction risks. This must be clarified in dose-ranging and chronic toxicity studies. Current preclinical evidence has shown that they are of low toxicity, but clinical studies in humans remain lacking. Clinical relevance requires bridging preclinical pharmacology with human trials. Anti-inflammatory, cardioprotective, and hepatoprotective effects have been shown in animal models and a human study. The challenges in the drug development of alpinetin and pinocembrin are the regulatory hurdles for botanical drugs and standardization of extraction, purification, and quality control. Moreover, drug–drug interaction risks via CYP450 and transporter inhibition must be managed. These aspects strengthen the therapeutic potential of alpinetin and pinocembrin, accelerating their progression from preclinical promise to clinically approved botanical medicines.

## Figures and Tables

**Table 1:** Comments on pharmacokinetic parameters (ADME) of alpinetin and pinocembrin.

Absorption and Distribution			
Pharmacokinetics Parameters	Alpinetin	Pinocembrin	Comment
Assessment analytical method	UHPLC-ESI-MS/MS, micellar electrophoresis	UPLC-ESI-MS/MS	These methods are highly sensitive compared to HPLC
Dosage for rats	5 mg/kg (oral, cardamom extract)	40 mg/kg of PCBG	Administration at different dosages
C_max_ (maximum plasma concentration)	385.633 ± 91.192 ng/mL	109.0 ng/mL	Alpinetin has a higher C_max_, greater systemic exposure, distributes widely, and has rapid clearance, but pinocembrin has a longer T_1/2_ (half-life).
T_1/2_ (half-life)	1.5784 ± 0.239 h	2.5 ± 0.0 h	
AUC (o-t) (area under the dosage curve)	911.723 ± 59.208 ng/mL·h	137.6 ng/mL·h	
Vz/F (apparent volume of distribution)	24.295 ± 6.858 L/kg	12.3 L/kg	
CLz/F (apparent clearance)	10.6834 ± 0.684 L/h/kg	3.4 L/h/kg	
**Metabolism**			
Assessment analytical method	UHPLC-TOF-MS	HPLC-DAD	UHPLC-TOF-MS is more sensitive than HPLC-DAD
Main metabolites	Prototypes, glucuronic acid conjugates, phenolic acid metabolites	Sulfonated and glucuronidated conjugate metabolites	Glucuronic acid conjugates as major metabolites
CYP450 interaction	Limit CYP-mediated biotransformation; selective inhibition of CYP1A2 and CYP3A4	Inhibit CYP3A4 and CYP2D6	Influence different cytochrome enzymes
Drug–drug interaction	Inhibit CYP3A4/CYP1A2-metabolized drugs	Inhibit CYP3A4, CYP2D6, and drug transporters, hOATP1A2 and hOATP2B1	The drug–drug interaction of both may decrease the blood concentration through CYP enzymes
Clinical implications	Dosage adjustment needed when co-administered with CYP1A2 or CYP3A4	Drug–drug interactions because of CYP and transporter inhibition	Manage the dosage and avoid drug–drug interaction
**Excretion**			
Phase II metabolism	Extensive glucuronidation	Glucuronidation	Rely on conjugation for clearance
Major metabolite(s)	Single major glucuronide metabolite	Glucuronide conjugates	Different metabolite profiles
UGT enzymes involved	UGT1A3, UGT1A1, UGT1A9, UGT2B15, UGT1A10		Involve the same enzymes
Excretion pathway	Transporter-mediated excretion (bile/urine)	Mainly fecal excretion	Clear alpinetin via bile or urine; pinocembrin is predominantly excreted via feces
**Conclusion**			
Based on the above information, alpinetin demonstrated faster absorption, higher systemic exposure, and bile/urine clearance, whereas pinocembrin showed slower clearance, longer half-life, and predominant fecal excretion.			

**Table 2:** Mechanisms of pharmacological actions of alpinetin and pinocembrin.

Pharmacological Actions		Flavonoids	Experimental Model	Rodent Strain (In Vivo/In Vitro)/ Administration Route/ Dose/Time	Mechanisms	References
Anti- inflammatory	Respiratory System	Alpinetin/Pinocembrin	allergic asthma/ allergic airway	BALB/c mice (in vivo) Intraperitoneal injection 10, 20, 40 mg/kg Once daily during OVA challenge phase EC_50_/IC_50_: not reported	PI3K/AKT/NF-κB, HO-1	[63,64]
		Alpinetin	COPD	Sprague–Dawley rats (in vivo) Oral gavage 25, 50, 100 mg/kg Once daily for 8 weeks following COPD induction EC_50_/IC_50_: not reported	PI3K/Akt/NF-κB STAT3/PI3K/Akt	[66]
		Pinocembrin	Lung injury or inflammation	C57BL/6 mice (in vivo) Oral gavage 20, 40 mg/kg Once daily during the induction/challenge period EC_50_/IC_50_: not reported	TLR4-NF-κB-NLRP3	[67]
	Digestive System	Alpinetin	Ulcerative colitis	C57BL/6 mice (in vivo) Oral gavage 20, 50, 100 mg/kg Once daily during DSS exposure EC_50_/IC_50_: not reported	① STAT3/IL-6 ② TLR4 and NLRP3 ③ AhR/Suv39h1/TSC2/mTORC1 ④ miR-302/DNMT-1/CREB	[74,78,81,82]
		Pinocembrin	Ulcerative colitis	C57BL/6 mice (in vivo) Oral gavage 20, 40, 80 mg/kg 7 days concurrent with DSS exposure EC_50_/IC_50_: not reported	TLR4/MD2/NF-κB	[83]
		Alpinetin	Pancreatitis	Sprague–Dawley rats (in vivo) Intraperitoneal injection 25, 50, 100 mg/kg Administered after induction of pancreatitis EC_50_/IC_50_: not reported	Aquaporin-1 regulation p38/ERK1/2 signaling modulation	[84]
		Pinocembrin	Pancreatitis	Sprague–Dawley rats (in vivo) Intraperitoneal injection 25, 50, 100 mg/kg Administered after induction of pancreatitis EC_50_/IC_50_: not reported	TLR4/NF-κB/NLRP3 miR-34a-5p/SIRT1/Nrf2/HO-1	[85]
	Reproductive System	Alpinetin	Mastitis	BALB/c mice (in vivo) Intraperitoneal injection 25, 50, 100 mg/kg Administered after LPS challenge EC_50_/IC_50_: not reported	TLR4/NF-κB	[88]
		Alpinetin	Endometritis	BALB/c mice (in vivo) Intraperitoneal injection 25, 50, 100 mg/kg Administered after LPS challenge EC_50_/IC_50_: not reported	TLR4/NF-κB PPAR-γ/NF-κB	[90]
	Locomotor System	Alpinetin	Osteoarthritis	C57BL/6 mice (in vivo) Intraperitoneal injection 25, 50, 100 mg/kg Administered daily after OA induction EC_50_/IC_50_: not reported	NF-κB/ERK	[94]
		Pinocembrin	Osteoarthritis	C57BL/6 mice (in vivo) Intraperitoneal injection 20, 40, 80 mg/kg Daily administration after OA induction EC_50_/IC_50_: not reported	NF-κB pathway inhibition	[97]
		Alpinetin	Foot inflammation	BALB/c mice (in vivo) Intraperitoneal injection 20, 40, 80 mg/kg Administered after carrageenan challenge EC_50_/IC_50_: not reported	PPARγ/NF-κB	[99]
		Pinocembrin	Rheumatoid arthritis	BALB/c mice (in vivo) Oral gavage 25, 50, 100 mg/kg Daily administration after arthritis induction EC_50_/IC_50_: not reported	Sox4/Stat3	[100]
	Cardiovascular System	Alpinetin	Acute myocardial infarction	Sprague–Dawley rats (in vivo) Intraperitoneal injection 25, 50, 100 mg/kg Administered after AMI induction EC_50_/IC_50_: not reported	TLR4/MyD88/NF-κB	[101]
		Pinocembrin	Post-infarct heart failure	C57BL/6 mice (in vivo) Oral gavage 20, 40, 80 mg/kg Daily administration after infarction EC_50_/IC_50_: not reported	Nrf2/HO-1	[102]
Anti- tumor	Gastrointestinal Cancer	Alpinetin	Gastric cancer	AGS and N87 (in vitro) 10 to 100 μM Cells treated for 24 to 48 h EC_50_: not reported IC_50_: 40 to 60 μM	Mitochondria-dependent endogenous apoptosis pathway	[103]
		Pinocembrin	Gastric cancer	AGS (in vitro) 10 to 100 μM Cells treated for 24 to 48 h EC_50_: not reported IC_50_: 40 to 60 μM	NF-κB signaling suppression miR-34a-5p modulation	[104]
		Alpinetin	Pancreatic cancer	BxPC-3 (in vitro) 10 to 100 μM Cells treated for 24 to 48 h EC_50_: not reported IC_50_: 40 to 60 μM	Bcl-2, XIAP, caspases	[110]
		Pinocembrin	Pancreatic cancer	Panc-1 (in vitro) 10 to 100 μM Cells treated for 24 to 48 h EC_50_: not reported IC_50_: 40 to 60 μM	NF-κB/ERK	[111]
	Gynecological Tumors	Alpinetin	Ovarian cancer	SKOV3 (in vitro) 10 to 100 μM Cells treated for 24 to 48 h EC_50_: not reported IC_50_: 40 to 60 μM	STAT3	[112]
		Pinocembrin	Ovarian cancer	SKOV3 and A2780 (in vitro) 10 to 100 μM Cells treated for 24 to 48 h EC_50_: not reported IC_50_: 40 to 60 μM	Epithelial–mesenchymal transition regulation, GABAB receptor pathway	[113]
		Alpinetin	Breast cancer	MDA-MB-231, 4T1, MCF-7 (in vitro) 10 to 100 μM Cells treated for 24 to 48 h EC_50_: not reported IC_50_: 40 to 60 μM	ROS/NF-κB/HIF-1α	[114]
		Pinocembrin	Breast cancer	MCF-7 (in vitro) 10 to 100 μM Cells treated for 24 to 48 h EC_50_: not reported IC_50_: 40 to 60 μM	PI3K/AKT	[115]
Cardiovascular Protection	Anti-Apoptosis	Alpinetin	Cardiomyocyte apoptosis model	Sprague–Dawley rats (in vivo) Intraperitoneal injection 25, 50, 100 mg/kg Administered prior to hypoxia/reoxygenation challenge EC_50_/IC_50_: not reported	PKC/ERK pathway Caspase-3, Bcl-2	[116,117]
		Pinocembrin	Pulmonary arterial hypertension model	Rats (in vivo) Review article	Rho A/ROCK	[118]
	Vasodilatation	Alpinetin	Mesenteric artery	Sprague–Dawley rats (in vivo) 1 to 100 μM (applied to isolated aortic rings) Acute exposure during organ bath experiments	① NO-mediated endothelium-dependent relaxation ② Endothelium-independent relaxation ③ PKC-dependent contraction	[124]
		Pinocembrin	Chronic ischemic heart failure	Sprague–Dawley rats (in vivo) Oral gavage 20, 40, 80 mg/kg Daily treatment after establishment of chronic heart failure EC_50_/IC_50_: not reported	Ion channel regulation	[125]
Liver and Kidney Protection		Alpinetin	Non-alcoholic fatty liver disease	Sprague–Dawley rats (in vivo) Oral gavage 25, 50, 100 mg/kg Daily treatment during high-fat diet feeding (several weeks) EC_50_/IC_50_: not reported	SOD1/Nrf-2/HO-1, TLR4/NF-κB	[138]
		Pinocembrin	High-fat diet-mediated non-alcoholic fatty liver	Sprague–Dawley rats (in vivo) Oral gavage 20, 40, 80 mg/kg Daily treatment during high-fat diet feeding (several weeks) EC_50_/IC_50_: not reported	Nrf2/HO-1/NF-κB	[139]
		Alpinetin	Liver and kidney injury	Male BALB/c mice (in vivo) Intraperitoneal injection 25, 50, 100 mg/kg Alpinetin administered prior to LPS challenge EC_50_/IC_50_: not reported	TLR4/NF-κB	[140,141]
		Alpinetin	Liver fibrosis	Sprague–Dawley rats (in vivo) Oral gavage 25, 50, 100 mg/kg Daily treatment during CCl_4_ exposure EC_50_/IC_50_: not reported	Nrf2/HO-1 NLRP3	[142]
		Pinocembrin	Liver injury	Male BALB/c mice (in vivo) Oral gavage 20, 40, 80 mg/kg Daily treatment following cisplatin exposure EC_50_/IC_50_: not reported	NF-κB/MAPK	[143]
		Pinocembrin	Kidney injury	HK-2 cells (in vitro) 10 to 100 μM Cells treated for 24 to 48 h EC_50_: not reported IC_50_: 40 to 60 μM	NF-κB Nrf2/HO-1	[144]
		Alpinetin	Hepatic ischemia–reperfusion injury	Male C57BL/6 mice (in vivo) Intraperitoneal injection 25, 50, 100 mg/kg Alpinetin administered prior to ischemia/reperfusion challenge EC_50_/IC_50_: not reported	NF-κB/MAPK	[143]
		Pinocembrin	Hepatic ischemia–reperfusion injury	Male C57BL/6 mice (in vivo) Oral gavage 20, 40, 80 mg/kg Administered prior to ischemia/reperfusion challenge EC_50_/IC_50_: not reported	HMGB1/TLR4	[144]
Other Effects		Alpinetin	Antibacterial activity	*Helicobacter pylori*, *Staphylococcus aureus*, *Staphylococcus epidermidis*, *Escherichia coli* (in vitro) 10 to 100 μM EC_50_/IC_50_: not reported	Drp1/HK1/NLRP3	[145]
		Pinocembrin	Antibacterial activity	*Aeromonas hydrophila* (in vitro) 10 to 100 μM EC_50_/IC_50_: not reported	Protein and DNA metabolism	[49]

**Table 3:** Relative efficacy, pharmacological advantages, and translational relevance of alpinetin and pinocembrin.

	Alpinetin	Pinocembrin	Differences
Relative efficacy	Higher systemic exposure with C_max_ 385.6 ng/mL 2–3-fold reduction in pro-inflammatory cytokines Strong anti-inflammatory, hepatoprotective, and anti-tumor efficacy	Lower systemic exposure with C_max_ 109 ng/mL 1.8-fold increase in antioxidant enzyme activity 2–4-fold lower minimum inhibitory concentrations (MICs) against Gram-positive bacteria	Alpinetin possesses systemic anti-inflammatory and multi-organ protection Pinocembrin is stronger in antibacterial potency
Pharmacological advantages	Faster absorption and higher plasma exposure Selective CYP1A2 inhibition and limited CYP-mediated metabolism Demonstrates therapeutic activity in the respiratory, digestive, cardiovascular, hepatic, and renal systems	Longer half-life with 2.5 h Inhibits CYP3A4, CYP2D6, and transporters Excellent blood–brain barrier penetration and mitochondrial protection	Alpinetin displays systemic versatility Pinocembrin offers longer persistence despite lower systemic exposure
Translational relevance	Broad therapeutic potential across multiple organ systems Requires formulation optimization to overcome bioavailability	Promise in neurological disorders, particularly ischemic stroke and Alzheimer’s disease	Alpinetin possesses multi-organ pharmacological activity but is still in preliminary translational stages Pinocembrin is more suitable for neurological applications

**Table 4:** Structured quality assessment of alpinetin and pinocembrin.

Item (s)	Alpinetin	Pinocembrin	Risk Assessment
Randomization	Rarely reported in animal studies; allocation methods are unclear	Rarely reported; allocation procedures are not described	High
Blinding	No evidence of blinding in pharmacokinetic or pharmacodynamic experiments	No blinding reported in antioxidant or signaling pathway studies	High
Sample size	Small-cohort studies with a limited number of rats	Similar to alpinetin	Moderate
Target	Cytokine reduction, NF-κB/MAPK modulation; clearly reported but heterogeneous	Antioxidant enzyme activity, Nrf2/HO-1 signaling; clearly reported but diverse	Moderate
Reproducibility	Pharmacokinetic parameters (C_max_, AUC, clearance) are reported; some methods are insufficient	Pharmacokinetic data are reported (PCBG studies), but methodological detail is limited	Moderate
Finding	Preclinical reports only	Preclinical reports only	High

## Data Availability

The data and materials used in this study are entirely based on publicly available datasets and information obtained through literature reviews.

## Abbreviations

The following abbreviations are used in this manuscript:

ADME	Absorption, Distribution, Metabolism, Excretion
AhR	Aryl Hydrocarbon Receptor
ALT	Alanine Aminotransferase
AMI	Acute Myocardial Infarction
APD	Action Potential Duration
AST	Aspartate Aminotransferase
AUC (0–t)	Area Under the Plasma Concentration–Time Curve from Time Zero to Time t
BALF	Bronchoalveolar Lavage Fluid
BCRP	Breast Cancer Resistance Protein
CHRM3	Muscarinic Acetylcholine Receptor M3
Clint	Intrinsic Clearance
CLz/F	Apparent Oral Clearance During the Terminal Phase
CNKI	China National Knowledge Infrastructure
CYP450	Cytochrome P450
CYP1A2	Cytochrome P450 1A2
CYP2A6	Cytochrome P450 2A6
CYP2C8	Cytochrome P450 2C8
CYP2C9	Cytochrome P450 2C9
CYP2D6	Cytochrome P450 2D6
CYP2E1	Cytochrome P450 2E1
CYP3A4	Cytochrome P450 3A4
C_max_	Maximum Plasma Concentration
COPD	Chronic Obstructive Pulmonary Disease
CREB	cAMP Response Element-Binding Protein
CX40/CX43	Connexin 40/Connexin 43
DSS	Dextran Sodium Sulfate
ER Stress	Endoplasmic Reticulum Stress
ESI	Electrospray Ionization
FDA	Food and Drug Administration
GSRS	Global Substance Registration System
HIF-1α	Hypoxia-Inducible Factor 1 Alpha
HPLC	High-Performance Liquid Chromatography
HPLC-DAD	High-Performance Liquid Chromatography–Diode Array Detection
HMGB1	High-Mobility Group Box 1
HAS	Human Serum Albumin
HO-1	Heme Oxygenase-1
ICAM-1	Intercellular Adhesion Molecule 1
IC50	Half Maximal Inhibitory Concentration
IL-1	Interleukin-1
IPF	Idiopathic Pulmonary Fibrosis
IR	Ischemia–Reperfusion
JAK2	Janus Kinase 2
MAPK	Mitogen-Activated Protein Kinase
MBC	Minimum Bactericidal Concentration
MD2	Myeloid Differentiation Protein 2
MIC	Minimum Inhibitory Concentration
MPO	Myeloperoxidase
mTORC1	Mechanistic Target of Rapamycin Complex 1
NAFLD	Non-Alcoholic Fatty Liver Disease
NASH	Non-Alcoholic Steatohepatitis
NF-κB	Nuclear Factor Kappa-Light-Chain-Enhancer of Activated B Cells
NGF	Nerve Growth Factor
NLRP3	NOD-, LRR-, and Pyrin Domain-Containing Protein 3 Inflammasome
Nrf2	Nuclear Factor Erythroid 2-Related Factor 2
OVA	Ovalbumin
PCBG	Pinocembrin-7-O-β-D-glucoside
PI3K/Akt	Phosphoinositide 3-Kinase/Protein Kinase B
PPAR-γ	Peroxisome Proliferator-Activated Receptor Gamma
PRISMA	Preferred Reporting Items for Systematic Reviews and Meta-Analyses
pHIMs	Pooled Human Intestine Microsomes
pHLMs	Pooled Human Liver Microsomes
ROS	Reactive Oxygen Species
SIRT1	Sirtuin 1
STAT3	Signal Transducer and Activator of Transcription 3
TCM	Traditional Chinese medicine
T1/2	Elimination Half-Life
TLR4	Toll-Like Receptor 4
TOF-MS	Time-of-Flight Mass Spectrometry
UGT	UDP-glucuronosyltransferase
UPLC-ESI-MS/MS	Ultra-Performance Liquid Chromatography–Electrospray Ionization–Tandem Mass Spectrometry
UHPLC-ESI-MS/MS	Ultra-High-Performance Liquid Chromatography–Electrospray Ionization–Tandem Mass Spectrometry
UHPLC-TOF-MS	Ultra-High-Performance Liquid Chromatography–Time-of-Flight Mass Spectrometry
Vz/F	Apparent Volume of Distribution During the Terminal Phase after Oral Administration
